# Suppressyn localization and dynamic expression patterns in primary human tissues support a physiologic role in human placentation

**DOI:** 10.1038/s41598-019-55933-x

**Published:** 2019-12-20

**Authors:** Jun Sugimoto, Danny J. Schust, Tadatsugu Kinjo, Yoichi Aoki, Yoshihiro Jinno, Yoshiki Kudo

**Affiliations:** 1 University of the Ryukyus, Graduate School of Medicine, Department of Molecular Biology, Okinawa, 903-0215 Japan; 2 University of the Ryukyus, Graduate School of Medicine, Department of Obstetrics and Gynecology, Okinawa, 903-0215 Japan; 3 University of Missouri, Department of Obstetrics, Gynecology and Women’s Health, Columbia, MO 65201 USA; 4 Hiroshima University, Department of Obstetrics and Gynecology, Hiroshima, 734-8553 Japan

**Keywords:** Differentiation, Endocrine reproductive disorders

## Abstract

We previously identified suppressyn (SUPYN), a placental protein that negatively regulates the cell fusion essential for trophoblast syncytialization via binding to the trophoblast receptor for syncytin-1, ASCT2, and hypothesized that SUPYN may thereby regulate cell-cell fusion in the placenta. Here, we redefine *in vivo* SUPYN localization using specific monoclonal antibodies in a rare early placental sample, showing SUPYN localization in villous and extravillous trophoblast subtypes, the decidua and even in placental debris in the maternal vasculature. In human trophoblast cell lines, we show SUPYN alters ASCT2 glycosylation within the secretory pathway and that this binding is associated with inhibition of cell fusion. Using newly-optimized trophoblast isolation protocols that allow tracking of *ex vivo* cell fusion, we present transcription and translation dynamics of fusion-related proteins over 96 hours in culture and the effects of changes in ambient oxygen levels on these processes. We report converse syncytin-1 and SUPYN transcriptional and translational responses to surrounding oxygen concentrations that suggest both are important in the effects of hypoxia and hyperoxia on placental syncytialization. Our results suggest that SUPYN’s anti-fusogenic properties may be exerted at several sites in the maternal body and its dysregulation may be associated with diseases of abnormal placentation.

## Introduction

There are several types of human trophoblast cells, each with fairly well-characterized localization and function at the maternal-fetal interface. Human placental villi from early pregnancy are covered by a specialized continuous, multinucleated cell layer that physically separates maternal and fetal circulations but allows for efficient gas and nutrient exchange and hormone production. This cell layer or syncytiotrophoblast is formed by the fusion of progenitor cytotrophoblast cells that lie just below the syncytiotrophoblast^1^. While cell fusion is essential to the development of a functional placenta, it must be tightly controlled and overly robust or deficient fusion have been associated with several pregnancy disorders^2^. The key to initiating trophoblast cell fusion likely resides in the villous cytotrophoblast cells, which generally reside in a discontinuous monolayer just beneath the syncytiotrophoblast of floating and all but the tips of anchoring placental villi^3^. At the tips of anchoring villi, cytotrophoblast cells grow to contact the maternal decidua as part of the cytotrophoblast cell column (CCC). Here, cytotrophoblast of the CCC differentiate into extravillous cytotrophoblast cells (EVTs) and invade across the maternal decidua as interstitial EVT, or along, across and into the maternal spiral arteries as endovascular EVTs. Endovascular EVTs replace cells within the maternal spiral artery, including the vascular endothelium, and thereby optimize vascular flow to the fetus^4^. Spiral artery segments that have been appropriately remodeled by endovascular EVTs are poorly responsive to maternal vascular signals and better able to buffer the fetus from adverse changes in maternal blood supply to the maternal-fetal interface^5–7^.

Formation of the syncytiotrophoblast involves cell fusion or syncytialization, a process common in viral infections but fairly unusual in most normal human tissues. It therefore may not be surprising that two important factors known to be involved in placental syncytialization are derived from viral envelope proteins. Syncytin-1 (SYN1)^8^ and syncytin-2 (SYN2)^9^ are truncated envelope (env) proteins from two distinct and unrelated human endogenous retroviruses (HERVs) that would have originally allowed for viral fusion with target host cells. Both retroviruses are thought to have infected host germ cells many millions of years ago. Both thereby became endogenized and able to be inherited in Mendelian fashion^10^. Many such HERV sequences can be found in the human genome (up to 8% of the total genome), but the vast majority have undergone significant mutations that have made them either non-functional or relegated them to a new but unknown function. A select few, however, have been co-opted for known physiologic function and the fusion-inducing function of SYN1 and SYN2 appear to have been maintained under selective pressure in a tissue specific fashion during the evolution of the human placenta^11,12^. Interestingly, this cooptation of viral fusogens into the placenta has been a decidedly successful strategy, as an ever-increasing group of species has been shown to use virally-derived env proteins during placental development. However, the originating viruses for most are quite diverse and unrelated and although the protein products have similar function, there are no truly orthologous retrovirally-derived placental fusogens among species^13^. SYN1, the translation product of the HERV sequence ERVWE1, is the prototype for placenta-specific expression and physiologic utilization of an endogenized viral fusogen. Mediating SYN1 fusion effects in the human placenta is its receptor, the neutral amino acid transporter, Alanine, Serine, Cysteine Transporter 2 ASCT 2 (aka SLC1A5: solute carrier family 1 member 5), that, in addition to the placenta, is expressed in almost all non-neural tissues^14,15^. Placental fusogenic specificity is therefore exerted by SYN1 gene expression patterns. SYN1 gene products are present throughout pregnancy in all trophoblast cell subtypes, both villous and extravillous, although protein expression is most robust in syncytiotrophoblast^16^.

We previously described a new placental HERV-derived protein with involvement in cell-cell fusion events. Unlike SYN1 and SYN2, suppressyn (SUPYN) inhibits syncytialization *in vitro* in trophoblast cell lines^17^. These anti-fusogenic effects were specific to SYN1- but not SYN2-mediated syncytialization. We demonstrated that SUPYN protein products were present in villous and extravillous trophoblast cells in placental specimens from the first and third trimesters using a polyclonal antibody. Since SUPYN could be detected both intracellularly and in a secreted form in cultured trophoblast cell models and both forms bound directly to the SYN1 receptor ASCT2, we hypothesized that SUPYN might inhibit SYN1-mediated fusion via paracrine and/or autocrine pathways. The aims of this study were threefold: (1) to better define the placental and decidual localization of HERV-related placental fusogens, anti-fusogens and their receptors *in vivo*, (2) to better understand the mechanisms underlying SUPYN’s antifusogenic activities and (3) to begin to define a functional link between SUPYN and abnormal placental development. More specifically, we hypothesized that SUPYN may exert its effects in the placental villi, in the maternal decidua and possibly in the maternal periphery and determined specific placental and decidial expression patterns for this and related molecules. We sought to verify the inhibitory effects of SUPYN on SYN1-mediated cell fusion using primary placental cells and addressed the possibility of intracellular mechanisms mediating SUPYN’s antifusogenic effects. Finally, we assessed the oxygen sensitivity of SUPYN transcription and translation using primary peri-term human trophoblast cells in culture to assess a potential role for SUPYN in the pathophysiology of placental developmental abnormalities that are reported to involve abnormal responses to changes in local oxygen tension^18^. Together, our results support an *in vivo* role for SUPYN in normal and abnormal placental development.

## Results

### Localization of human placental fusogens, antifusogens and their receptors *in vivo*

**Figure 1:**
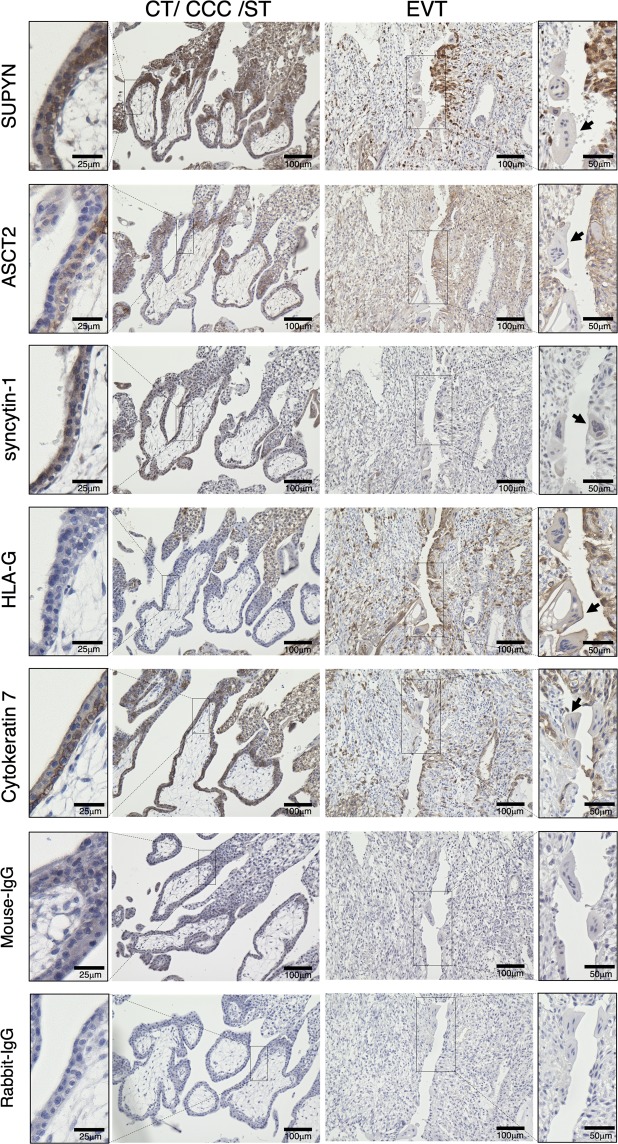
Localization of cell fusion related proteins in the first trimester placenta. Immunohistochemical analysis of a tissue sample from a woman at 7 weeks of gestation who underwent elective hysterectomy for cervical cancer with her pregnancy *in situ*. The left side shows the result of immunohistochemical staining for cytotrophoblast cell (CT), cytotrophoblast cell column (CCC), and syncytiotrophoblast (ST) areas and the right side shows extravillous cytotrophoblast cells (EVT). Arrows indicate syncytialized debris. The two bottom rows of images are of isotype control immunostaining. These panels use the same area of the specimens as depicted above. Scales for images are as indicated.

Using an optimized anti-SUPYN monoclonal antibody (clone: 3H6) (Supplementary Fig. S1) and a very rare tissue sample from a woman at 7 weeks of gestation who underwent elective hysterectomy for cervical cancer with her pregnancy *in situ*, we refined our understanding of SUPYN localization and confirmed prior descriptions of HLA-G, cytokeratin 7 (CK7) and known human placental fusogens and their partners in early gestation (Fig. 1). Because we had access to a specimen that could be sectioned through the implantation site and out through the entirety of the decidua and myometrium, we include IHC images that are limited to the placental/decidual interface (Fig. 1) as well as more global images that include deeper maternal uterine tissues (Supplementary Fig. S2). SUPYN protein was seen to be expressed in both villous and extravillous cytotrophoblast cells but had more limited expression in syncytiotrophoblast (Fig. 1). As expected, HLA-G was confirmed as a specific marker for EVT and the monolayer epithelial cell marker CK7 was present in cytotrophoblast, intermediate cytotrophoblast, EVT and endometrial glandular epithelium^19^. Minimal cross-reactive labeling was noted in control experiments using only mouse and rabbit isotype control antibodies (Fig. 1 and Supplementary Fig. S2). More specifically, SUPYN was detected in the progenitor cytotrophoblast cells lying just below the multinucleated syncytiotrophoblast at the villus surface, in the intermediate cytotrophoblast present at sites where anchoring villi attach to the maternal decidua and in the EVT leaving the anchoring villi to invade through the maternal decidual interstitium. Interestingly, SUPYN protein was strongly expressed at sites where the vascular endothelium has been replaced by EVT (as indicated by similar localization patterns for the EVT markers HLA-G and CK7) (Fig. 1). Since we had previously shown in co-immunoprecipitation experiments that SUPYN protein binds to the SYN1 receptor ASCT2, we assessed ASCT2 expression in these same specimens. Like SUPYN, ASCT2 expression in the placental villi was high in the cytotrophoblast layer, although unlike SUPYN, its expression in the syncytiotrophoblast layer was essentially undetectable. In stark contrast, SYN1 was expressed more strongly in syncytiotrophoblast than in cytotrophoblast.

We were fortunate to identify several multinucleated placental fragments (Fig. 1, arrows) within a maternal vascular structure bisecting the specimen. We believe these are fragments of syncytialized trophoblast debris within the maternal spiral arteries. These structures were strongly positive for the EVT marker, HLA-G. When compared to any surrounding negative decidual cells, the syncytialized debris was also positive, albeit weakly, for SYN1 and possibly SUPYN and ASCT2.

Elsewhere within the decidua, we detected cells strongly expressing HLA-G and SUPYN that have morphologic and localization features consistent with EVT, When compared to negative control specimens using isotype matched antibody, ASCT2 appears to label most cells within the decidua, albeit with varying intensities.

### Effects of SUPYN on the glycosylation of ASCT2

**Figure 2:**
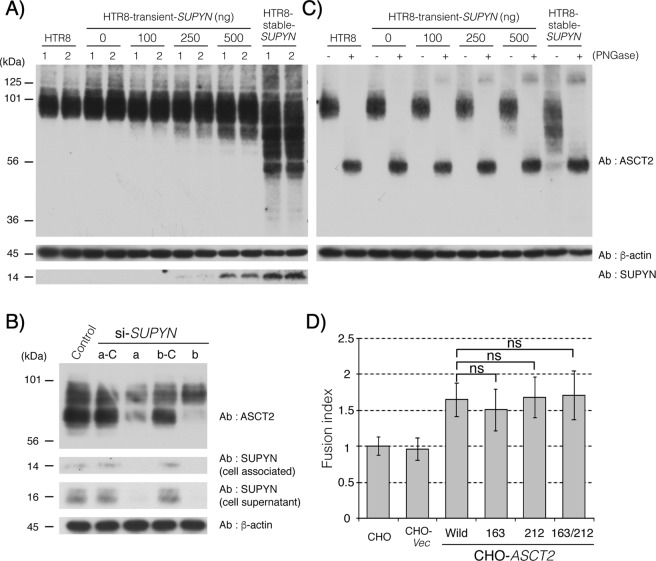
Association between SUPYN and ASCT2 induces glycosylation changes. (**A**) ASCT 2 [Alanine, Serine, Cysteine Transporter 2, the syncytin- 1 (SYN1) receptor] western blot analysis after transiently expressing suppressyn (SUPYN) in HTR8 cells. The numbers 1 and 2 represent technical replicates. (**B**) Structural changes in ASCT2 protein upon knockdown of SUPYN in BeWo cells using SUPYN-specific siRNAs. Control-vector alone; a and b are two distinct siRNA constructs. The a-C and b-C are their respective scrambled siRNA controls. (**C**) Effects of PNGase treatment of HTR8 cells on ASCT2 glycans that are altered in response to transient expression of SUPYN. The + and − refer to the presence and absence of peptide: N-glycosidase (PNGase), respectively. (**D**) Changes in SYN1-induced fusion of CHO cells transiently expressing wild-type ASCT2 and three ASCT2 proteins with glycosylation site mutations. CHO–fusion in parent CHO cells; CHO-*Vec*–CHO cells transfected with vector only; CHO-*ASCT2*–CHO cells transfected with a vector driving the expression of wildtype (Wild) ASCT2 or ASCT2 constructs with mutations affecting the glycosylation sites at aa 163 (163), 212 (212) or both the 163 and 212 (163/212) positions. Statistical analysis was performed using the Mann Whitney U test with Bonferroni correction. Values represent means ± SDs (n = 3). ns: not significant compared with the wild type ASCT2. Immunoblots have been cropped for clarity, conciseness and comparison. The full-length immunoblot for (**B**) is included as Supplementary Fig. S8 in the Supplementary Materials and Methods.

Using co-immunoprecipitation experiments, we previously reported that both cell-associated and secreted SUPYN bound directly to the SYN1 receptor, ASCT2. However, cell-associated SUPYN fully inhibited cell fusion while secreted SUPYN caused incomplete but detectable (approximately 30%) inhibition. To further clarify the nature and effects of cell-associated binding, we transiently transfected HTR8 cells with a vector driving flag-tagged SUPYN expression. In this system, the presence of increasing amounts of SUPYN was associated with the development of an elongated smear of smaller molecular weight forms of endogenous ASCT2 in western immunoblots (Fig. 2A), suggesting protein degradation, ubiquitination or possibly abnormalities in the maturation of endogenous ASCT2. We further leveraged BeWo cells and their robust SUPYN expression^17^ to perform a converse experiment in which SUPYN expression was inhibited using SUPYN-specific siRNAs and their scrambled control siRNAs. As shown in Fig. 2B, exposure to two independent siRNAs substantially decreased the amounts of the lower molecular weight forms of ASCT2 that were present in wildtype and control siRNA treated SUPYN-expressing BeWo cells when compared to the larger forms (Fig. 2B, lanes 3 vs. 2 and 5 vs. 4). ASCT2 is a known glycoprotein with two N-linked glycans. We further evaluated the identities of the low molecular weight ASCT2 forms seen in the presence of SUPYN by treating HTR8 cells that transiently or stably expressed SUPYN (as in Fig. 2C) with peptide N glycanase, which removes N-linked glycans. Removal of N glycans completely abrogated the intermediate low molecular weight forms of ASCT2 on western blot (Fig. 2C, even numbered lanes), with all detectable ASCT2 forms collapsing into a single band with a molecular weight 56 kDa, the predicted size of the ASCT 2 protein backbone.

### Effects of changes in ASCT2 glycosylation on the antifusogenic effects of SUPYN on SYN1-mediated cell-cell fusion

To further delineate possible functional effects of impaired glycan maturation on ASCT2-mediated cell fusion, we created a group of cell lines that could be induced to fuse in the presence of SYN1 but that also expressed ASCT2 forms containing mutations affecting glycosylation. Three expression vectors were created that changed N(Asn) to H(His) at either or both of the ASCT2 N-glycosylation sites (N163H, N212H, N163H/N212H). These expression vectors were transiently transfected into CHO cells that normally express neither wildtype ASCT2 nor SYN1 and the presence of the mutated forms of ASCT2 protein detected using western immunoblotting (Supplementary Fig. S3A). Twenty-four hours after transfection with the ASCT2 mutant and control vectors, the CHO cells were transiently co-transfected with a vector driving the expression of fusion-inducing SYN1. CHO cell fusion was evaluated 24 hours later using flow cytometry to calculate fusion indices. (Fig. 2D). Mutation of either or both of the ASCT2 N-glycosyation sites had no effect on SYN1-induced cell fusion when compared to wildtype controls (Fig. 2D, Supplementary Fig. S3B). Although intracellular binding of SUPYN to ASCT2 alters the glycan maturation of ASCT2, these results would indicate that it is the binding itself, rather than effects on glycosylation that inhibit SYN1/ASCT2 mediated syncytialization.

### Fusion-related transcriptional and translational dynamics in peri-term primary human trophoblast cells *ex vivo*

**Figure 3:**
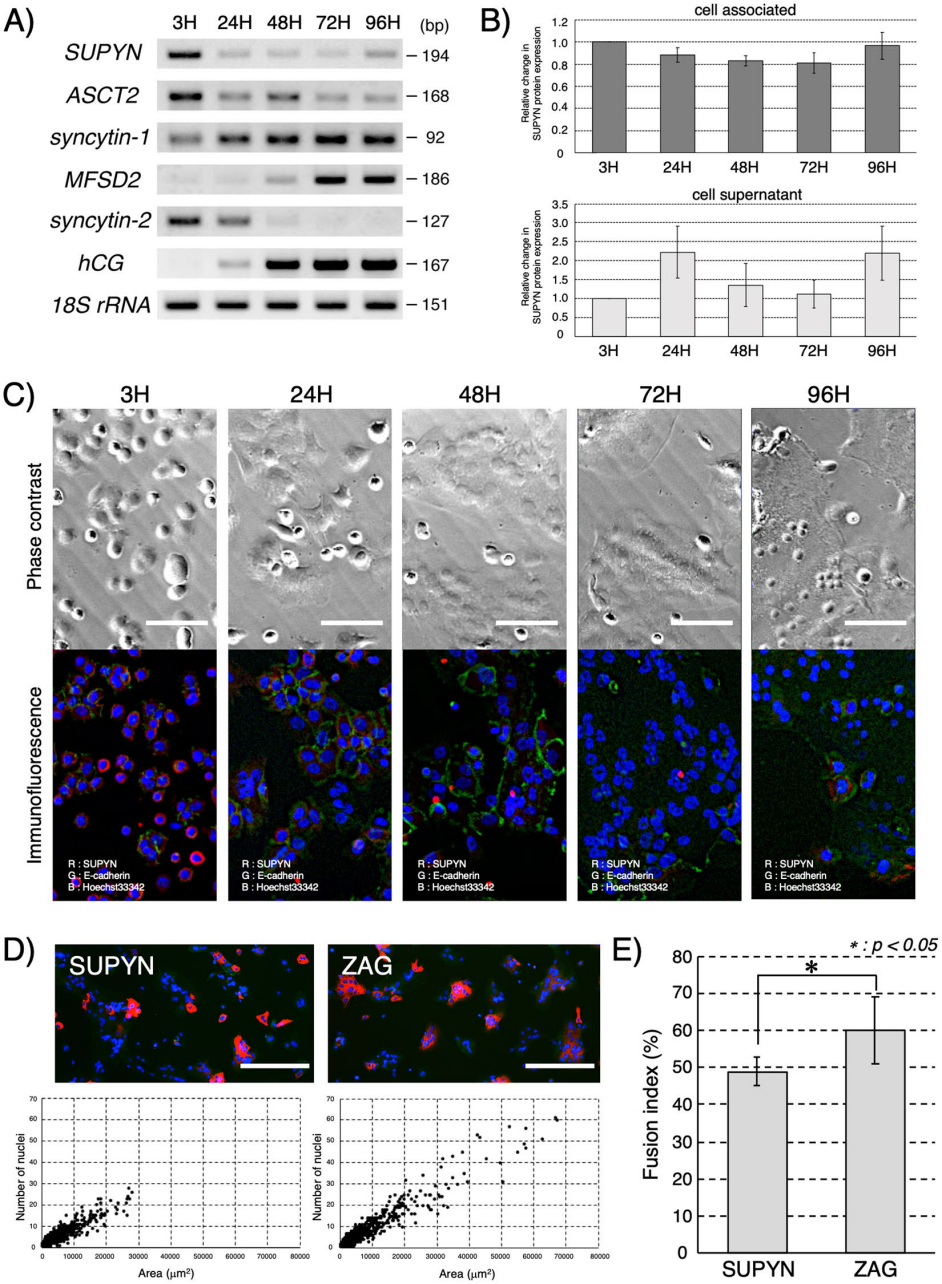
Cell fusion and expression of placental fusion-related proteins and hormones in primary spontaneously syncytializing peri-term human cytotrophoblast cells with and without forced SUPYN expression. (**A**) Expression of fusion-associated genes in primary trophoblast cells using semi-quantitive RT-PCR. Gels have been cropped for clarity, conciseness and comparison. Full length gel images for Fig. 3A are included as Supplementary Fig. S9 in the Supplementary Materials and Methods. (**B**) Detection of SUPYN protein in primary trophoblast cell cultures by a SUPYN-specific ELISA. Data depict relative fold change compared with 3H timepoint (cell-associated values normalized to 3H cell-associated baseline and cell supernatants to 3H cell supernatant). Upper panel, cell associated SUPYN; Lower panel, SUPYN in supernatants. Experiments were performed in duplicate using 4 independent placenta samples. Values represent means ± SDs (n = 4). (**C**) Time course of SUPYN protein expression in cultured primary peri-term cytotrophoblast cells. The upper panels show phase contrast images at each time point after plating, the lower panels an overlay fluorescence immunocytochemical analysis of an adjacent field [SUPYN protein (red), E-cadherin (green) and nucleus (blue)]. Scale bar = 50 micrometers. (**D**) Cellular morphologic changes after transient FLAG-tagged SUPYN (SUPYN) and control, FLAG-tagged ZAG (ZAG) overexpression in cultured primary peri-term cytotrophoblast cells. Anti-FLAG antibody (red) detects SUPYN-expressing cells and anti-ZO-1 (green) detects cell boundaries. Nuclei are stained blue. Expression of SUPYN and ZAG were detected by an anti-FLAG antibody, and the number of nuclei in SUPYN- or ZAG-expressing cells were plotted. (**E**) The number of nuclei in SUPYN- or ZAG-expressing cells was counted and fusion indices were calculated. Experiments were performed three independent times. Values represent means ± SDs (n = 3). Paired t-tests were used for statistical comparisons. Statistical significance (*p < 0.05). *SUPYN*–suppressyn; *ASCT2*–Alanine, Serine, Cysteine Transporter 2, the syncytin 1 receptor; *MFSD2*–major facilitator superfamily domain-containing protein 2, the syncytin 2 receptor; *hCG*–human chorionic gonadotropin; *ZO-1*–zona occludens-1.

We utilized our newly optimized protocol for isolation of cytotrophoblast from human peri-term placenta^20,21^ to follow RNA and protein expression dynamics for a variety of molecules involved in cell-cell fusion during the process of spontaneous syncytialization of primary cytotrophoblast cells and to verify SUPYN’s anti-fusogenic activities in primary trophoblast cells *ex vivo*. Gene expression patterns were examined by RT-PCR (Fig. 3A and Supplementary Fig. S5). Verifying previous reports using cells also cultured in a 5% CO_2_/20% ambient O_2_ atmosphere^22^, we noted that SYN1 expression increases progressively as cells syncytialize spontaneously over the first 72 hours of *ex vivo* culture, but then stabilizes at the 96 hour timepoint. In contrast, transcription of the SYN1 receptor, ASCT2, is high 3 hours after cell isolation but progressively decreases over the 96 hour culture period. These patterns are consistent with placental protein localization patterns in our 7 week tissue sample (Fig. 1). Transcription of SYN2 RNA peaks at 24 hours in culture and remains low while that of its receptor, major facilitator superfamily domain-containing protein 2 (MFSD2), is initiated by 24–48 hours and continues to increase through 96 hours, again showing a reciprocal expression pattern of a HERV-derived placental fusogen and its receptor. An expected increase in the transcription of the syncytiotrophoblast marker human chorionic gonadotropin (hCG) across the syncytialization process (first detected at 24 hours in culture) was also noted. We show for the first time in primary trophoblast cells *ex vivo* that the *SUPYN* gene is highly expressed in cytotrophoblast cells immediately after isolation. *SUPYN* transcription then declines rapidly as fusion progresses but is detected again at the 96 hour timepoint. This phenomenon was confirmed at the protein level in these primary trophoblast cultures for both cell-associated and soluble SUPYN by immunocytochemistry and the results of a newly established SUPYN-specific ELISA assay, respectively (Fig. 3B,C). Likely due to the time lag involved in the secretory process, the peak level of SUPYN protein in cell supernatants is delayed by about 24 hours when compared to cell-associated SUPYN. In concordance with our transcription data, levels of secreted SUPYN increased again at 96 hours. Subcellular localization dynamics of SUPYN protein during the process of spontaneous cell-cell fusion was visualized by fluorescence immunocytochemistry under standard 5% CO_2_/ambient O_2_ conditions (Fig. 3C). At the beginning of culture, expression of SUPYN is noted in the cytoplasm of almost every cell. At this time, E-cadherin can be detected outlining the cell boundaries of single, unfused, mononuclear cytotrophoblast cells. By 24 hours, cells have begun to cluster but cytoplasmic SUPYN and cell surface E-cadherin continue to characterize these mostly unfused cells. By 48 hours, cell-cell fusion has been established and multinucleated syncytialized areas are outlined by E-cadherin. At this point, very little SUPYN protein can be detected. Interestingly, by 72 hours, even though syncytialization continues, we begin to detect SUPYN reappearance and by 96 hours, we can see the reappearance of unfused single cells and detect cytoplasmic SUPYN in both independent cells and multinucleated syncytial patches. The physiologic relevance of these dynamics in our *ex vivo* experiments is not presently known, but it is enticing to speculate on the possibility that we are seeing cytotrophoblast progenitor cell survival during *ex vivo* syncytialization.

### Effects of forced SUPYN expression on peri-term primary human trophoblast cell syncytialization *ex vivo*

The ability of freshly isolated primary cytotrophoblast to spontaneously syncytialize in culture combined with the rapid down regulation of SUPYN expression upon culture provides an opportunity to assess the anti-fusogenic effects of SUPYN in primary cells *ex vivo* under conditions of forced SUPYN expression. Immediately after isolation, primary cytotrophoblast cells were transiently transfected by electroporation with a pCAG vector driving the expression of SUPYN fused in frame to a FLAG sequence, as previously described^17^. Cells were then cultured at 37 °C in 5% CO_2_/room air for 96 hours. As a control, replicate primary cytotrophoblast cells were transiently transfected with the same vector driving the expression of ZAG (Alpha-2-Glycoprotein 1, Zinc-Binding), a secretory protein with size similar to SUPYN but a physiologic function unrelated to cell fusion. Confirming prior results in trophoblast and non-trophoblast cell lines, the presence of SUPYN inhibited spontaneous fusion in these primary cells when compared to ZAG-expressing controls as shown by: 1) IHC (Fig. 3D upper), 2) convergence of SUPYN-expressing cells when nuclei per cell area is plotted (Fig. 3D lower) and 3) significantly reduced auto-calculated cell fusion indices (*p* < 0.05; Fig. 3E) that were 4) confirmed using standard manual counting methods (Supplementary Fig. S4A,B)

### Effects of ambient oxygen conditions on cell-cell fusion and fusion-related transcriptional and translational dynamics in primary peri-term human trophoblast cells *ex vivo*

**Figure 4:**
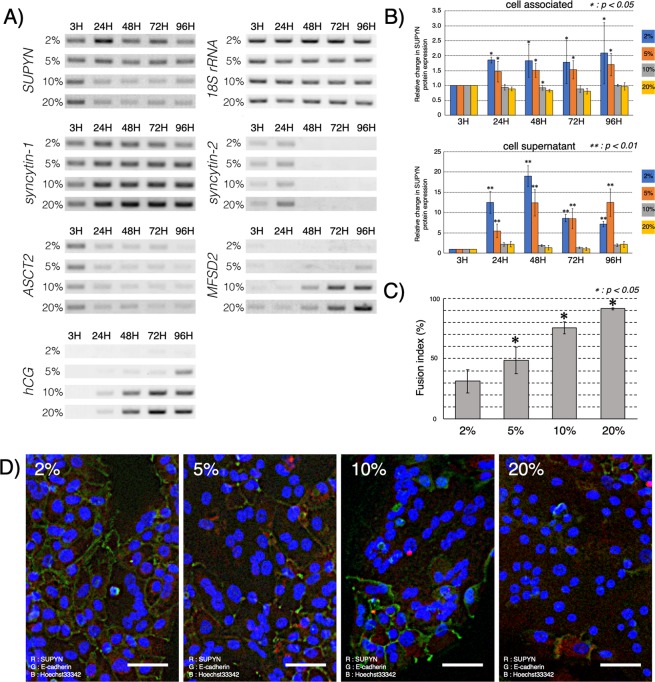
Effects of ambient O_2_ on cell fusion and expression of fusion–related proteins in primary human peri-term villous cytotrophoblast cell cultures. (**A**) Expression of fusion-associated genes using semi-quantitative RT-PCR under various O_2_ conditions. Gels have been cropped exactly as in Fig. 3A for clarity, conciseness and comparison. (**B**) Detection of SUPYN protein by a SUPYN-specific ELISA. Data represent relative fold change compared with 3 H timepoint. Experiments were performed using 4 independent placenta samples, each in duplicate. Statistical analysis was performed using the Mann Whitney U test with Bonferroni correction. Values represent means ± SDs (n = 4). Statistical significance (*p < 0.05, **p < 0.01) compared with the 20% O_2_ condition at each time point. (**C**) Fusion indices for cells grown under 2% to 20% O_2_ conditions. 4 independent microscopic views were used to calculate fusion rates and paired-t-tests were used for statistical analysis. Values represent means ± SDs (n = 4) Statistical significance (*p < 0.05) compared with the 2% O_2_ condition. (**D**) Expression of SUPYN protein in primary cytotrophoblast cells cultured for 96 H in various O_2_ environments. Fluorescence immunocytochemistry analyses show an overlay with SUPYN protein (red), E-cadherin (green) and nucleus (blue). The scale bar = 50 micrometers. *ASCT2*–Alanine, Serine, Cysteine Transporter 2, the syncytin 1 receptor; *MFSD2*–major facilitator superfamily domain-containing protein 2, the syncytin 2 receptor; *hCG*–human chorionic gonadotropin.

Placental syncytialization in humans and other mammals is known to be sensitive to ambient oxygen concentrations and placental fusogen partners and their regulators, such as SYN1, ASCT2 and glial cells missing transcription factor 1 (GCM1) have been shown to respond to hypoxia and hyperoxia in trophoblast cell lines *in vitro* and primary trophoblast cells *ex vivo*^22–25^. We evaluated SUPYN response to different ambient oxygen concentrations at the RNA and protein levels by culturing primary cytotrophoblast cells isolated from peri-term placentas in several different (2%, 5%, 10% and 20% ambient) O_2_ environments. In primary cells that spontaneously express SUPYN, SUPYN transcription and translation/secretion increased when grown under reduced ambient oxygen concentrations when compared to those exposed to 10% O_2_ conditions and room air (Fig. 4A,B and Supplementary Fig. S5). There were minimal changes in SUPYN transcription noted under 5% vs 2% low O_2_ conditions and results from 10% O_2_ conditions mimicked those in 20% O_2_ conditions. In contrast, both low O_2_ conditions inhibited the transcription of the syncytialization-dependent gene, hCG, reflecting inhibition of cell fusion. Expression of the SYN2 receptor, MFSD2, was also lower in low O_2_ compared to higher O_2_ environments. Oxygen concentration had minimal effects on the expression of ASCT2 and SYN2 mRNA (Fig. 4A and Supplementary Fig. S5). Consistent with the transcriptional expression pattern changes for SYN1, MFSD2, SUPYN and the syncytiotrophoblast secretory hormone hCG, cell-fusion increased significantly as ambient O_2_ concentrations increased (Fig. 4C,D).

## Discussion

In this study, we had three primary aims: (1) to better define the placental and decidual localization of HERV-related placental fusogens, anti-fusogens and their receptors *in vivo*, (2) to better understand the mechanisms underlying SUPYN’s antifusogenic activities and (3) to begin to define a functional link between SUPYN and abnormal placental development.

We conducted a detailed immunohistochemical characterization of SUPYN localization and of the protein expression patterns of other known HERV-derived placental fusogens and their receptors in a rare specimen from early human gestation that included the entire uterus removed for medical reasons with a 7 week gestation *in situ*. While most protein staining simply verified prior reports, using an optimized monoclonal antibody against SUPYN, we here show that SUPYN protein levels are evident in cytotrophoblast cells (villous and extravillous) and while detectable in the syncytiotrophoblast, it is at lower levels than in the adjacent villous cytotrophoblast cells *in vivo*. We believe there are several reasons why the latter results differed from our prior publication in which we used a polyclonal anti-SUPYN antibody and reported detection of SUPYN in both the cytotrophoblast and syncytiotrophoblast layers of chorionic villi^17^. The most likely reasons for the discrepancy are the use of a more specific and more importantly, a more sensitive anti-SUPYN reagent. It is also possible that a small amount of cytoplasmic SUPYN may remain in ST after cell fusion and this causes faint SUPYN signals to be detected in multinucleated ST. Importantly, our present optimized results demonstrate preferential expression of SUPYN in unfused trophoblast cells and support its anti-fusogenic activities.

Tissue expression patterns for the fusogens, antifusogen and fusogen receptors studied here are striking. ASCT2, the SYN1 receptor, and SUPYN, the putative inhibitor of SYN1-ASCT2 mediated syncytialization are detected on the same cells–villous cytotrophoblast and EVT–suggesting these molecules may be co-expressed to allow for control of ASCT2 interactions with SYN1. Staining of maternal decidual cells for ASCT2 has been reported^26^ and is also noted in our specimen. In contrast, SYN1 was detected in both trophoblast layers of the villous placenta, although expression appears much more robust in ST. In agreement with prior reports, we detected weak expression of SYN1 in villous and extravillous cytotrophoblast^16^. Combining IHC results with an examination of expression dynamics for SUPYN, SYN1, SYN2, ASCT2 and MFSD2 in spontaneously syncytializing *ex vivo* primary cytotrophoblast cultures, we are able to hypothesize a role for SUPYN in inhibiting cell fusion at several sites throughout the maternal-fetal interface and even in the maternal periphery.

IHC of the 7 week implantation site demonstrated robust expression of SUPYN in EVT at the tips of anchoring villi, in those invading through the decidual interstitium and myometrium and in the endovascular EVT, which also express HLA-G. The latter suggests that SUPYN may be involved in remodeling the maternal spiral arteries and successively, the etiology of diseases of poor placentation such as pregnancy-induced hypertension and preeclampsia, a possibility deserving future detailed study. Fortuitously, the clinical sample available for this study contained several fragments of syncytialized cells trapped within the large maternal vessel that bisected the specimen. This debris was labeled by the HLA-G specific antibody (Fig. 1 arrowheads), indicating that it is the product of EVT fusion and suggesting a potential role for EVT-expressed SUPYN in controlling the production of syncytial debris resulting from fusion of SYN1- and ASCT2- positive EVT in addition to the debris shed at the surface of floating villi (see below). Since increases in syncytial debris have been seen in the periphery of women with preeclampsia^27,28^, we believe there is a need to further study these findings in women affected by this and related disorders.

We hypothesize that at the surface of the floating villi, SUPYN helps to control pro-fusogenic interactions between surface-expressed SYN1 on syncytiotrophoblast (and possibly cytotrophoblast) and ASCT2 expressed on the surface of all villous cytotrophoblast. This could help to regulate both deficient syncytialization and overly robust syncytialization with resultant shedding of ST debris, some of which will bear SYN1^29^. Since SUPYN can be cell-associated or secreted and both forms appear to inhibit fusion (see^17^ and below), SUPYN derived from villous cytotrophoblast could also inhibit fusion of syncytiotrophoblast debris with maternal local and peripheral ASCT2-expressing somatic cells. Finally, our *ex vivo* data on the transcriptional dynamics of the placental fusogens in spontaneously fusing primary cytotrophoblast from peri-term deliveries was again striking in that expression of SUPYN and ASCT2 mirrored each other and along with SYN2, were active only early in the fusion process. In contrast, expression of SYN1 and the SYN2 receptor, MFSD2, began to increase later in the fusion process and did not overlap with the temporal expression of their individual pro-fusogenic partners. This too supports the concept that SUPYN may be important in inhibiting ASCT2-mediated events. Interestingly both RT-PCR and immunocytochemistry revealed that late in the syncytialization process, primary peri-term trophoblast cells will begin to once again express SUPYN RNA and protein. (Fig. 3A,B) In immunocytochemistry experiments, cytoplasmic and perinuclear SUPYN protein, which was essentially nondetectable at 48 hours, begins to be detected again by 72 hours. By 96 hours in culture, single, non-fused mononuclear SUPYN-expressing cells can also be seen (Fig. 3C). The mechanisms underlying these dynamic expression and localization patterns remain unknown and their elucidation is likely to shed light on normal and abnormal placental development.

We identified a potential mechanism by which SUPYN may functionally control SYN1-mediated trophoblast fusion at the level of the SYN1 receptor, ASCT2 (Fig. 2). In the process of defining the direct binding of SUPYN to ASCT2 through co-immunoprecipitation assays using SUPYN overexpressing and knockdown trophoblast cell line models, we noted a marked change in the appearance of ASCT2 when run on PAGE gels for western immunoblotting. We performed a series of experiments in SUPYN-expressing and non-expressing trophoblast cell lines to show that the changes seen in western immunoblotting were the result of alterations in the N-linked glycans on ASCT2. While this effect most likely results from direct binding of SUPYN to ASCT2 within the cell secretory pathway and subsequent alteration in ASCT2 glycan maturation, mutation of the N-glycosylation sites on ASCT2 did not affect its effects on SYN1-mediated cell fusion^15^. This indicates that the binding of SUPYN to ASCT2 is sufficient to mediate its antifusogenic activities; however, we cannot definitively state that immature surface glycans are not also involved. To this point, we observed a nearly 100% inhibition of fusion in cultured HTR8 trophoblast cells stably expressing SUPYN in prior work^17^ and secreted SUPYN could also inhibit cell fusion events, introducing the possibility that SUPYN inhibits ASCT2-mediated, SYN1-induced trophoblast fusion through several mechanisms and that the predominant mechanism may be site specific (e.g., within the co-expressing villous CT and EVT vs. in the maternal periphery).

We here explored for the first time the effects of ambient oxygen levels on SUPYN transcription. Preeclampsia is characterized by shallow placentation and resultant hypoxia-reoxygenation damage and several authors have assessed the protein and RNA dynamics of SYN1 and its receptor in *in vitro* and *ex vivo* models of placental hypoxia. Most have agreed that significant hypoxia decreases cell fusion, triggers GCM1 degradation and is associated with decreases in SYN1 and either decreased or unchanged ASCT2^22–25,30^. Disruptions in appropriate oxygen-responsive changes in fusogenic proteins have been hypothesized to be involved in the placental pathologies seen in women with preeclampsia and, indeed, SYN1 levels are lower in primary placental tissues from pregnancies affected by preeclampsia^25^. Using Real-time RT-PCR, Kudaka *et al*. reported in 2003 that SUPYN (*ERVH48-1 or HERV-Fb1*) expression is decreased in placentas from women who experienced pregnancy-induced hypertension or preeclampsia^31^. Here, we used primary cells to examine the *ex vivo* effects of a range of oxygen conditions on SUPYN expression. Since the oxygen content at the human maternal-fetal interface is quite hypoxic, particularly during the first trimester of pregnancy prior to the initiation of robust intervillous maternal blood perfusion^32,33^, 20% ambient O_2_ conditions would certainly be considered hyperoxic and likely expose the trophoblast cells to oxidative damage. The exact level of hypoxia at the maternal-fetal interface, however, has not been definitively established and it likely changes across early pregnancy as the placenta moves from a largely histiotrophic to a more generally hematotropic environment^32–34^. In primary cytotrophoblast cells, cell-associated and secreted SUPYN protein levels were lower in the presence of hyperoxia when compared to both of the low O_2_ conditions (Fig. 4B). Results under 10% O_2_ exposures generally mimicked those from 20% ambient O_2_ conditions. Interestingly, although both hypoxia and hyperoxia at the maternal-fetal interface have been purported to be involved in the pathogenesis of preeclampsia, only hyperoxic conditions have been reported^35–37^ to be associated with both preeclampsia and fetal growth restriction. We noted transcriptomic changes in SUPYN and some of the placental fusogens and their receptors when hyperoxic conditions were compared to more physiologic low O_2_ environments but much more subtle changes when comparing the two low O_2_ environments. Deficient SUPYN production in the presence of hyperoxia would be predicted to release its control on syncytialization at the surface of the chorionic villi, and potentially between EVT in the decidua, both increasing maternal exposure to syncytialized debris. It could also have detrimental effects on maternal spiral artery remodeling and both would be consistent with some of the pathologic findings in preeclampsia. Future experiments using specimens from women with and without disorders of abnormal placental development such as preeclampsia and fetal growth restriction are certainly warranted.

## Methods

### Placenta samples

During the development and expansion of our *in vitro* models for the study of SUPYN, a female subject at the Hiroshima University was diagnosed with cervical carcinoma and chose surgical treatment with hysterectomy. At the time of diagnosis, she was found to be in her first trimester of pregnancy. She consented to have her surgical specimen used for research purposes and uterus, cervix and the pregnancy tissues were removed *in toto* with the 7 week pregnancy *in situ*. These tissues were used for immunohistochemical analyses. Clinical samples used for *in vitro* primary trophoblast cultures were collected from otherwise discarded placentas from normal peri-term deliveries. All placentas were obtained from clinically-indicated caesarean section deliveries at 36–37 weeks of gestation. Indications included previous caesarean section or placenta previa. Other than placenta previa in some women, all enrolled subjects were healthy and had otherwise uncomplicated pregnancies. All collections were approved by the Ethical Committee for Human Genome Research of the University of the Ryukyus School of Medicine and the Hiroshima University and appropriate informed consent was obtained from all participants. All research was performed in accordance with relevant guidelines/regulations.

### Isolation and culture of primary villous trophoblast cells

To aid in experimental reproducibility of syncytialization in *in vitro* cultures and improve time management, we optimized methods for isolating and culturing primary trophoblast cells from human peri-term placentas^20,21^. Briefly, placentas were aseptically collected and immediately cooled with iced PBS. Villous tissues were obtained from biopsies that were at least 5 mm from both the amniotic and decidual surfaces. The blood vessels were removed from the villous tissue and isolated villous tissues were minced and washed with cold PBS. This mixture was then filtered through a 100 μm stainless steel sieve and pretreated in a cell dissociation solution (Trypsin 0.125% (10 × Trypsin:15090046: Thermo Fisher scientific, Waltham, MA, USA), DNase I 200 U (DR-1S: Worthington, Lakewood, NJ, USA)/HBSS Ca+, Mg+ (14025: Thermo Fisher scientific, Waltham, MA, USA) at room temperature for 5 minutes. The solution was again filtered through a 100 μm stainless steel sieve and approximately 10–20 g of wet volume tissue was placed in a 500 ml flask containing 50 ml of the same cell dissociation buffer with reciprocal shaking (90 rpm) at 37 °C. After one hour, the mixture was filtered through a 60 μm stainless steel sieve. Five ml of FBS was added to the solution and the extracted cells were recovered by centrifugation at 600 g × 10 min. The cell pellet was suspended in Buffer 1 (0.1% BSA/DPBS) and layered on the top of a Percoll gradient (17089102: GE, Chicago, USA) layered from 70% to 10% at 10% intervals in a 15 ml tube and centrifuged at 1500 g × 30 min. The target cytotrophoblast cell population (Supplementary Fig. S6A) was collected from each of the 40% and 50% layers and washed again with buffer 1. Isolated cells were counted, suspended at a concentration of 2 × 10^6^/ml in Trophoblast Medium (TM) (#7121:Sciencell, Carlsbad, CA, USA) and cultured in aliquots of 100 μl/well in 48 well plates at 37 °C in 5% CO_2_ and varying O_2_ concentrations as indicated in individual experiments (2%, 5%, 10% and 20%). Three hours after seeding, the plates were manually agitated and nonadherent cells (e.g., hematopoietic cells) removed by changing the medium. Fresh media was added and the cells were cultured for up to 96 hours at 37 °C, 5% CO_2_ and O_2_ as indicated with daily media changes.

Since this was a modified isolation protocol, we performed additional verification assays. The two isolated cell groups (40% and 50% layers; Supplementary Fig. S6A) were confirmed as trophoblast by their characteristic cellular morphology, conserved fusogenic potential in culture in the absence of exogenous stimulation (e.g.,without cAMP or forskolin) and expression of known trophoblast markers (Supplementary Fig. S6B). Those in the 50% fraction had minimal contamination by syncytial debris. The purity of these percoll-isolated primary cells was verified by flow cytometry (Supplementary Fig. S6B). The cells expressed the trophoblast marker CD49f (anti-very late antigen (VLA)-6 α chain)^38^ but not the epithelial cell/fibroblast marker, CD49a (anti-VLA-1)^39^. HLA-G was used to discriminate single cell EVT and HLA-ABC to detect contaminating hematopoietic cells. Primary cytotrophoblast cells were therefore identified as CD49f (+), CD49a (−), HLA-G (−) and HLA-ABC (−) and a purity of >90% was verified in multiple independent purification assessments. In this newly developed method for cytotrophoblast isolation from peri-term placentae, we found that HBSS containing Mg and Ca was critical as it helped to limit cell damage and optimized cell dissociation by trypsin and DNase activity. Prepared cells were purified from typical contaminants using a simple Percoll separation, making the protocol straightforward, effective and reproducible.

### ELISA

A SUPYN-specific monoclonal antibody (clone: 2J16) was dissolved in carbonate buffer (0.5 μg/ml) and used as the capture antibody. 100 μl of the capture antibody solution was placed into wells on a 96-well plate and incubated overnight at 4 °C. Plates were washed 5 times with PBST (PBS with 0.05% Tween 20) using a microplate washer (ImmunoWash 1575:BioRad, Hercules, CA, USA) and blocked with 0.5% BSA/PBST for 1 hour at room temperature. After washing, 100 μl of sample was dispensed into each well and incubated at 4 °C overnight. Plates were washed five times and HRP labeled anti-SUPYN monoclonal antibody (clone: 3H6) was used as a detection antibody at a concentration of 0.2 μg/ml. After 1 hour at room temperature, plates were washed 5 times and incubated with 100 μl of POD (ELISA POD Substrate TMB kit: 05298-80: nakalai tesque, Kyoto, Japan) for 7 minutes at RT. Color development was stopped with 100 μl of H_2_SO_4_ and absorbance measured at a wavelength of 450 nm by a microplate reader (TriStar Lb941:Berthold, Bad Wildbad, Germany). Experiments were performed using 4 independent placentas and two technical replicates per assay at each O_2_ condition. ELISAs were performed in duplicate for each sample. In preliminary experiments, dilution linearity was performed using an artificially synthesized 50aa SUPYN peptide (111–160) for ELISA assay validation (Supplementary Fig. S7A) and culture supernatants or lysates from those cancer-derived villous (BeWo, JAR), suppressyn-expressing and non-expressing (HTR8) trophoblast cell lines^40^, as well as the non-expressing endometrial cancer-derived cell line (Ishikawa) were used as positive and negative controls. (Supplementary Fig. S7B,C).

### Fusion inhibition in primary trophoblast cells

Cytotrophoblast cells isolated from peri-term placentas were washed with D-PBS and suspended in D-PBS at a concentration of 1 × 10^6^ cells/ml. Ten μg of plasmid was added to 0.5 ml (5 × 10^5^ cells) of cells and the solution incubated on ice for 10 minutes. The plasmid containing the *ERVH48-1* (suppressyn-encoding) ORF sequence with an in-frame flag sequence, and a ZAG (Alpha-2-Glycoprotein 1, Zinc-Binding: NM_001185) ORF sequence with an in-frame FLAG sequence in a pCAG vector was used. Using a Gene Pulser electroporation system (BioRad, Hercules, CA, USA), electroporation was carried out in a 0.4 cm cuvette at 250 V and 300 μF. After 5 minutes on ice, cells were suspended in 500 μl of TM medium and cultured on a 48-well plate. Six hours later, non-adherent cells were removed by medium exchange and adherent cells were cultured for an additional 96 hours at 37 degrees in 5% CO_2_. Cells were fixed with 4% paraformaldehyde for 15 minutes and exposed to the fluorescently-labelled anti-FLAG antibody or anti-ZO-1 antibody at 4 °C for 24 H. The multiple fluorescence images of an entire single well of a 48 well plate were captured (Flag antibody: Red, ZO-1 antibody: Green and Nucleus: Blue) by a Keyence microscope (BZ-X710; KEYENCE Japan, Osaka, Japan) and stitched together via the merge function. The areas of fused cells in control, ZAG stained with flag antibody (Red), were determined using the masking function of the Cell Hybrid counting system software. The masked areas were specified by comparing to ZO-1 stained (Green) fluorescence to delineate the outline of fused areas. The specified value for masking determined in the ZAG image was then applied to SUPYN transfected cell images and nuclei were counted in masked, fused cells measured under the parameters determined by the control ZAG images. Since masking was identical for ZAG and SUPYN bias was minimized and quantitation over an entire well limited image capture bias. Cells containing three or more nuclei were considered to be fused. All cells in a well were analyzed and the cell fusion rate calculated using three independent experimental replicates and the formula [(N − S)/T] × 100; N is the number of nuclei in a given syncytial patch, S is the number of syncytia, and T is the total number of nuclei counted. Keyence-generated results were validated using standard manual counting methods as shown in Supplementary Fig. S4A,B and described in detail in the Supplementary Materials.

Detailed descriptions of experimental methods using more standard approaches can be found in the Supplementary Materials and Methods.

## Supplementary information

Supplementary Data

## Data Availability

The data that support the findings of this study are available from the corresponding author upon reasonable request.
