# Supplementary material for: Suppressyn localization and dynamic expression patterns in primary human tissues support a physiologic role in human placentation
Source: Sci Rep. 2019 Dec 20;9:19502. doi: 10.1038/s41598-019-55933-x (PMC6925194; doi:10.1038/s41598-019-55933-x)
Supplement: Supplementary file 1 — Supplementary Data [file 41598_2019_55933_MOESM1_ESM.pdf]

# Suppressyn localization and dynamic expression patterns in primary human tissues support a physiologic role in human placentation.

Jun Sugimoto[1,4,+,*], Danny J Schust[3,+], Tadatsugu Kinjo[2], Yoichi Aoki[2], Yoshihiro Jinno[1], Yoshiki Kudo[4]

[1]University of the Ryukyus, Graduate School of Medicine, Department of Molecular Biology, Okinawa, 903-0215, Japan
[2]University of the Ryukyus, Graduate School of Medicine, Department of Obstetrics and Gynecology, Okinawa, 903-0215, Japan
[3]University of Missouri School of Medicine, Department of Obstetrics, Gynecology and Women's Health, Columbia, MO, 65201, USA
[4]Hiroshima University, Department of Obstetrics and Gynecology, Hiroshima, 734-8553, Japan

[*]Corresponding. jsokiaji1@gmail.com
[+]These authors contributed equally to this work

# Supplementary Materials and Methods

(RT-PCR)

Beginning 3 hours after placement in culture, primary cytotrophoblast cells were collected every 24 hours. A Qiagen RNeasy Plus kit (74134: Qiagen, Valencia, CA, USA) was used for total RNA isolation in accordance with the manufacturer's instructions. 200ng of the total RNA was subjected to reverse transcription using a ReverTraAce kit (FSK-101:Takara, Shiga, Japan) at 30°C for 10 minutes, 42°C for 60 minutes and 95°C for 5 minutes. The resultant mixture was diluted ten fold and 4 μl of the diluted product was subjected to PCR in a 10 μl volume (KAPATaq Extra HS Ready Mix with dye: KK3606 : Nippon Genetics, Tokyo, Japan). Reaction parameters were: 30 cycles of 94°C for 30 seconds, 60°C for 30 seconds, and 72°C for 30 seconds. PCR products were electrophoresed on a 1.5% agarose gel and visualized. Gels have been cropped for clarity, conciseness and comparison. Full length gel images for Figure 3A are included as Supplementary Figure S9 in this document. The primers used for RT-PCR are listed in Supplementary Table S2.

(Quantitative RT-PCR)

Six microliters of a ten fold diluted reverse transcription product was used for real time RT-PCR with SYBR Green I in 25μl total volume (Applied Biosystems *Power* SYBR Green Master Mix: 4368577: Thermo Fisher Scientific, Waltham, MA, USA ) The primers used for real-time PCR are listed in Supplementary Table S2. The analysis of relative gene expression data was performed using the $2^{-\Delta\Delta CT}$ method and represent relative fold change compared with the 3H timepoint.

(Fluorescence immunocytochemistry)

Primary cultured cells were fixed every 24 hours with 4% paraformaldehyde. Blocking was carried out with 5% normal serum/0.3% Triton X-100/PBS solution for 1 hour at room temperature. The primary antibody reaction was allowed to proceed overnight at 4°C with a combination of the polyclonal anti-suppressyn antibody and the monoclonal anti-E-cadherin antibody in antibody dilution buffer (1% BSA/0.3% Triton X-100/PBS solution). Slides were then washed three times with PBS for 5 minutes each. Appropriate secondary Alexa Fluor 488 goat anti-mouse IgG or Alexa Fluor 555 goat anti-rabbit IgG antibodies were added for 1 hour at room temperature. Antibody dilutions are listed in Supplementary Table S1. Nuclei were stained with Hoechst 33342 (0.5μg/ml). Flourescent signals were detected using a Fluorescent microscope unit (BZ-X710; KEYENCE Japan, Osaka, Japan).

(Immunohistochemistry)

Tissue blocks were prepared from formaldehyde-treated tissue using standard methods. Slides with 4 μm sections were heated at 60°C for 15 minutes and then standard deparaffinization with xylene was performed. Antigen retrieval was carried out using an electric kettle at 98°C for 40 minutes in 0.2 M citrate buffer at pH 6.0. Inactivation of endogenous peroxidase activity was performed in 0.3% $H_2O_2$/methanol at room temperature for 20 minutes. Sections were blocked with horse serum (for mouse antibodies) or donkey serum (for rabbit antibodies) at room temperature for 30 minutes. Primary antibodies were diluted as in Supplementary Table S1 and sections were incubated with primary antibodies overnight at 4°C. Slides were washed three times in PBS and exposed to biotinylated antibodies at room temperature for 30 minutes, then to the streptavidin-HRP antibody at room temperature for an additional 30 minutes (VECTORSTAIN Elite ABC HRP kit : PK-6102, PK-6101:VECTOR Laboratories, Burlingame, CA, USA or Streptavidin-Biotin Complex Peroxidase kit: 30462-30：nakalai tesque, Kyoto, Japan). Color development utilized DAB (Peroxidase Stain DAB kit: 25985-50：nakalai tesque, Kyoto, Japan) and hematoxylin staining was done in the standard fashion at room temperature for 5 minutes.

# Supplementary Materials and Methods

(Cell Lines)
The CHO (Chinese hamster ovary), BeWo and JAR cell lines were purchased from ATCC (Manassas,VA, USA) and HTR8 cells were a kind gift from Professor Charles Graham of the Department of Anatomy and Cell Biology at Queen's University, Kingston, ON, Canada. HTR8 cells are a frequently used transformed trophoblast cell line thought to best model EVT and known to express SUPYN at negligible levels. [17, 40] The endometrial Ishikawa cell line was generously supplied by Dr Susan Nagel at The University of Missouri. HTR8, Ishikawa and CHO cells were cultured in DMEM (041-29775:Fuji film, Tokyo, Japan) supplemented with 10% FBS (Fetal Bovine Serum:Gibco-Thermo Fisher scientific, Waltham, MA,USA); BeWo and JAR cells were cultured in Ham's F12 (087-08335:Fuji film, Tokyo, Japan) with 15% FBS.

(Transient gene expression and gene knockdown by siRNA)
HTR8 cells were adjusted to a cell number of 2 $x10^5$ cells/well in 12 well plates and cultured overnight (at 37°C and 5% $CO_2$ condition) to 70% confluence. The suppressyn gene, *ERVH48-1*, fused to a FLAG sequence was cloned into a mammalian expression vector (previously described as pCAG vector) [17]. HTR8 cells were transfected with 0 ng to 500 ng of vector in 1.5 μl of Lipofectamine 2000 (11668027: Thermo Fisher Scientific, Waltham, MA, USA). After two days of incubation at 37°C and 5% $CO_2$, cells were lysed in RIPA buffer for western blot analysis. Knockdown of the gene encoding suppressyn by siRNA using BeWo cells was performed as described previously [17]. In the ASCT2 mutant experiments, CHO cells were cultured in 6-well plates at $2x10^5$ cells/well and then transfected with 2μg of a variety of pMyc vectors as previously described [17] into which wildtype and mutant (163, 212, 163/212) ASCT2 sequences had been cloned. Transfection used 3 μl of lipofectamine 2000 (11668067:Thermo Fisher Scientific, Waltham, MA, USA) ). The numbers used to describe the ASCT2 mutants refer to the amino acid sites at which an N(Asp) had been changed to an H(His) by site-directed mutagenesis using the PrimeSTAR Mutagenesis Basal kit (R046R:Takara, Shiga, Japan) according to the manufacturer's protocol with oligonucleotides as described in Supplementary Table S2. After culturing for 24 hours at 37°C in 5% CO2, 1μg of the pFlag-SYN1 vector was co-transfected into each CHO cell line and the cells were cultured for an additional 24 hours at 37°C in 5% CO2. Cell fusion was assessed by flow cytometry as previously reported [17].

(Western immunoblotting and N-glycosidase treatment)
Equal amounts of protein were treated with N-glycosidase (P0704S:New England Biolabs, Ipswich, MA, USA) according to the manufacturers' recommendations. Proteins were then analyzed using standard PAGE and western immunoblotting (Figure 2C and Supplementary Figure S3A).

(Flow cytometry analyses)
Freshly isolated primary placental cells were cultured on plastic for 3 hours to remove nonadherent cells. Adherent cells were then washed with D-PBS and recovered using 0.05% trypsin for 5 minutes at 37°C. The collected cells were blocked with 1% BSA/PBS for 20 minutes and 3 μl of indicated antibodies (BioLegend, San Diego, CA, USA) were added to the cell suspension (Supplementary Table S1). After 30 minutes at 4°C, cells were washed twice in FACS buffer (0.1% BSA/PBS) and analyzed by FACSverse (BD, San Joes, CA, USA).

(Antibodies and Primers)
All antibodies are shown in Supplementary Table S1. Primers for gene cloning, mutagenesis, and RT-PCR are shown in Supplementary Table S2.

# Supplementary Materials and Methods

(Manual quantification of fusion indices)

Cell fusion was assessed using Keyence microscope and quantitated using cell fusion indices. The multiple fluorescence images of an entire single well of a 48 well plate were captured (Flag antibody : Red, ZO-1 antibody : Green and Nucleus : Blue ) by a Keyence microscope (BZ-X710; KEYENCE Japan, Osaka, Japan). The fusion index quantitates the percentage of fusion events in a cell population. Analyses were performed using 6 randomly-selected independent fields in 3 independent samples by two independent analysts blinded to the origin of the sample. The number of fused syncytial aggregates and the number of nuclei in each aggregate was counted manually and fusion indices were defined as [(N-S)/T] x 100. N is the number of nuclei in syncytia, S is the number of syncytia, and T is the total number of nuclei counted.

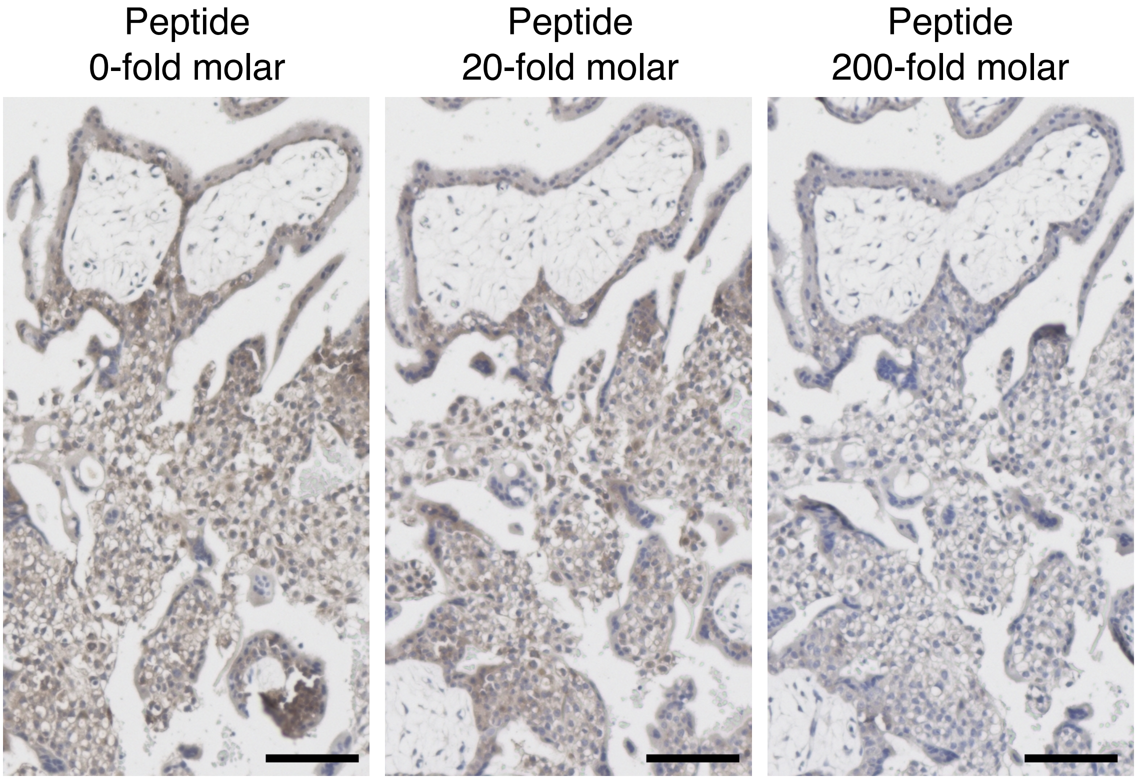


**Supplementary Figure S1. Monoclonal anti-suppressyn antibody specificity.**
The anti-suppressyn monoclonal antibody, 3H6, was incubated overnight with 0-, 20- and 200-fold molar excess of a blocking peptide designed based on our anti-suppressyn antibody antigen recognition epitope. This antibody was then used as the primary antibody for immunohistochemical analysis of first trimester placental samples as in Figure 1. The scale bar = 100 micrometers.

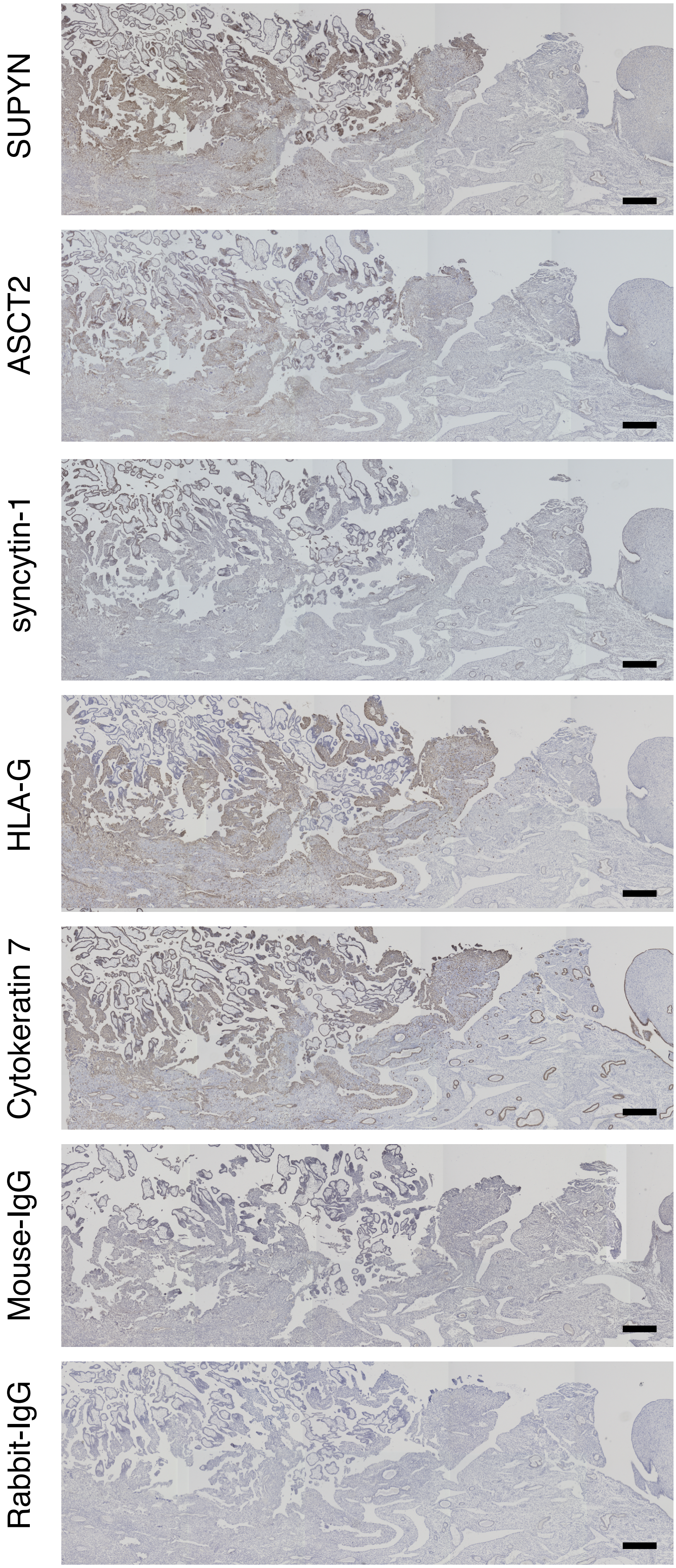


**Supplementary Figure S2**
**Immunohistochemical staining of cell fusion-associated proteins in a uterine sample with a 7 week gestation *in situ*.** Immunostaining for SUPYN, ASCT2, syncytin-1, HLA-G, cytokeratin 7 and mouse- and rabbit-IgG isotype controls were performed using samples containing placental villi, decidua, and myometrium. The scale bar = 500 micrometers.

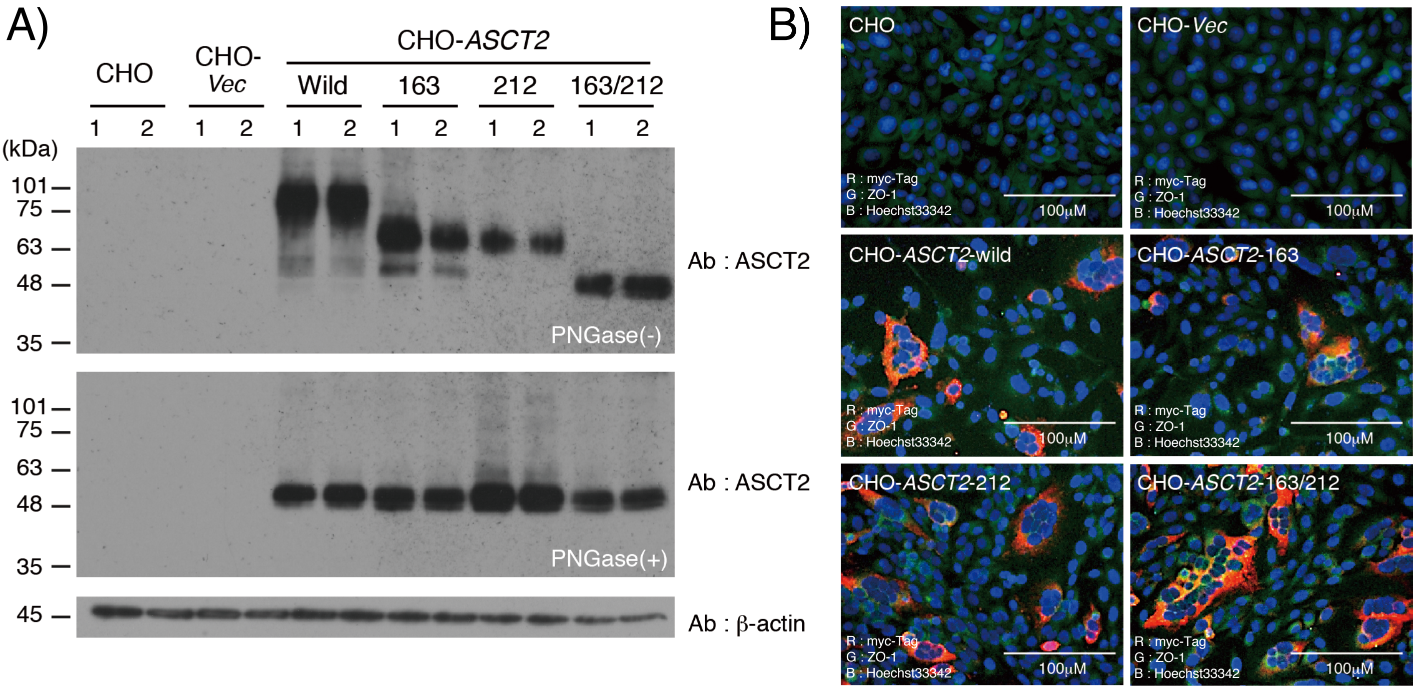


**Supplementary Figure S3. Expression of ASCT2 variants with N-glycosylation site mutations (Asp to His) at positions 163, 212 and both 163 and 212.**

A) Western immunoblotting demonstrates size changes in N-glycosylation site ASCT2 mutants that are abrogated upon PNGase treatment. The numbers 1 and 2 represent technical replicates. B) Induction of cell fusion by SYN1 in ASCT2 mutant cells. ASCT2-myc (red), ZO-1 (green), nucleus (blue). The scale bar = 100 micrometers. Immunoblots have been cropped for clarity, conciseness and comparison.

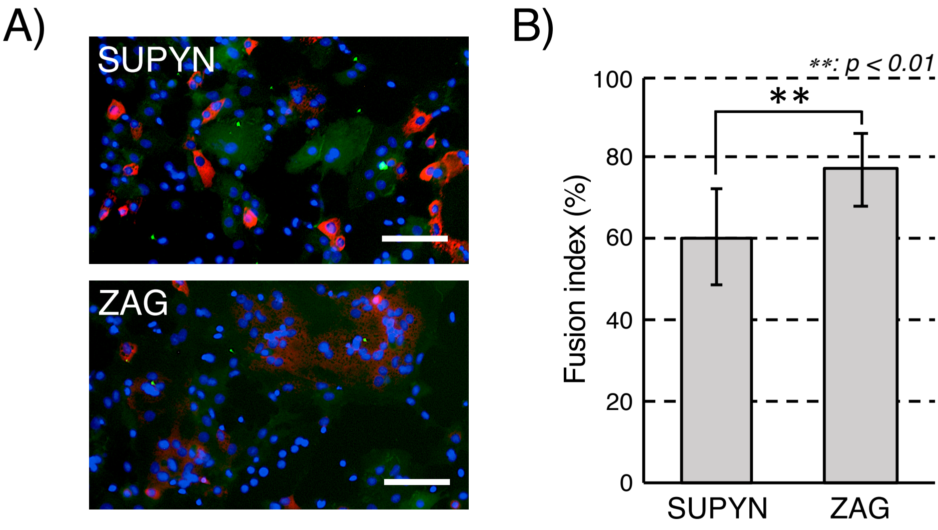


**Supplementary Figure S4. Cell fusion in primary spontaneously syncytializing peri-term human cytotrophoblast cells with and without forced SUPYN expression**

A) Cellular morphologic changes after transient FLAG-tagged SUPYN (SUPYN) and control, FLAG-tagged ZAG (ZAG) overexpression in cultured primary peri-term cytotrophoblast cells. Anti-FLAG antibody (red) detects SUPYN- (or ZAG-)expressing cells and anti-ZO-1 (green) detects cell boundaries. Nuclei are stained blue. The scale bar = 100 micrometers. B) The number of nuclei in SUPYN- or ZAG-expressing cells were counted manually and fusion indices were calculated. Experiments analyzed 6 independent fields in 3 independent samples and were performed by two independent blinded analysts. Values represent means ± SDs (n=6). Paired t-tests were used for statistical comparisons. A $p$ value below 0.01 (** : $p<0.01$) was considered significantly different.

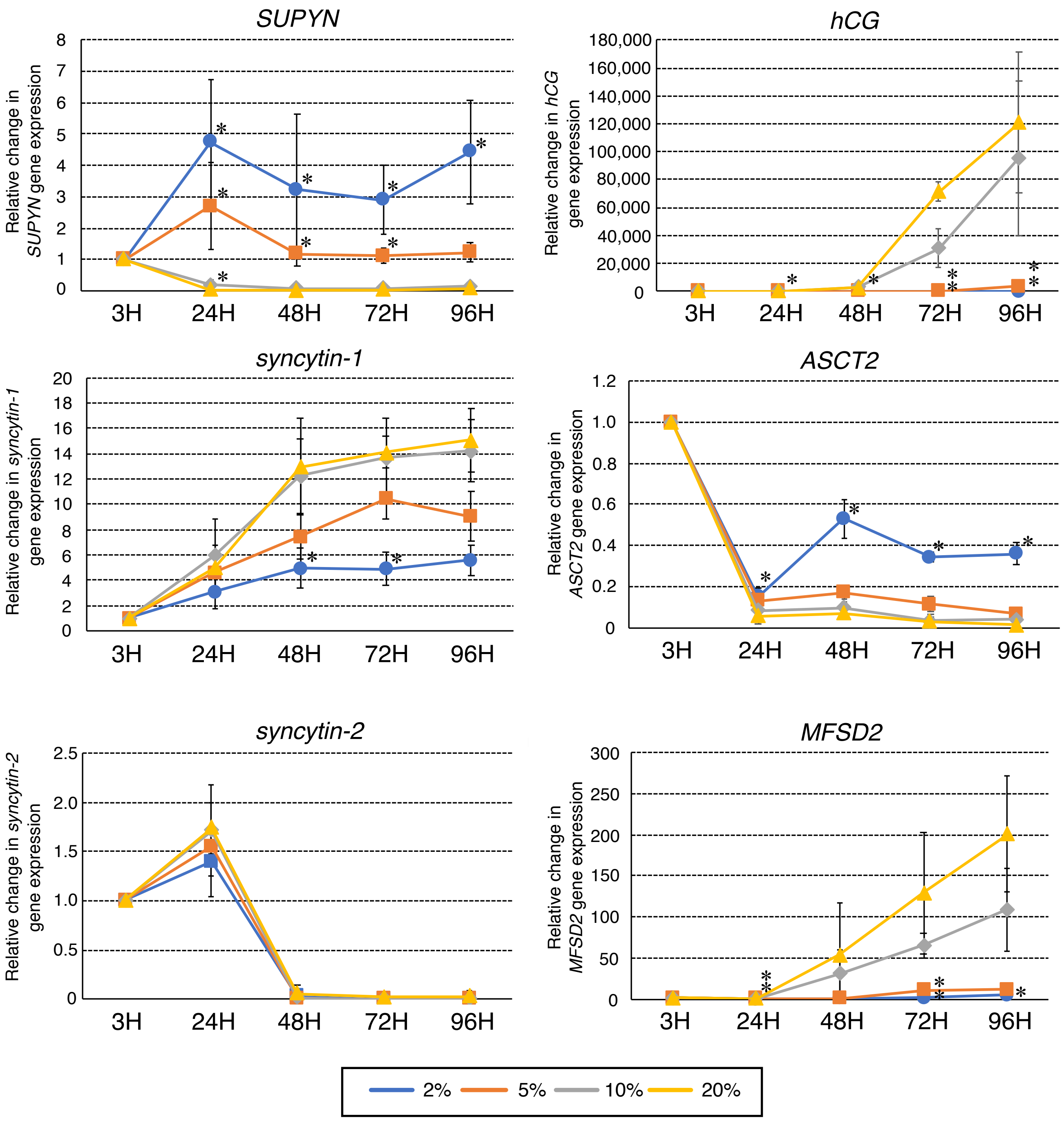


**Supplementary Figure S5. Temporal expression of fusion-associated genes using quantitative RT-PCR analyses under various $O_2$ conditions.**

Quantitative RT-PCR analyses were performed with samples from 4 independent placentas. Data were analyzed by $2^{-\Delta\Delta CT}$ methods and plotted relative fold change compared with the 3H time point and corrected for expression of the control gene (18S rRNA). Values represent means ± SDs (n = 4). Statistical analysis was performed using the Mann Whitney U-test. A *p* value below 0.05 (* : $p<0.05$) was considered significantly different compared with the 20% $O_2$ condition at each time point. *ASCT2*-- Alanine, Serine, Cysteine Transporter 2 , the syncytin-1 receptor; *MFSD2*-- major facilitator superfamily domain-containing protein 2, the syncytin-2 receptor; *hCG*-- human chorionic gonadotropin.

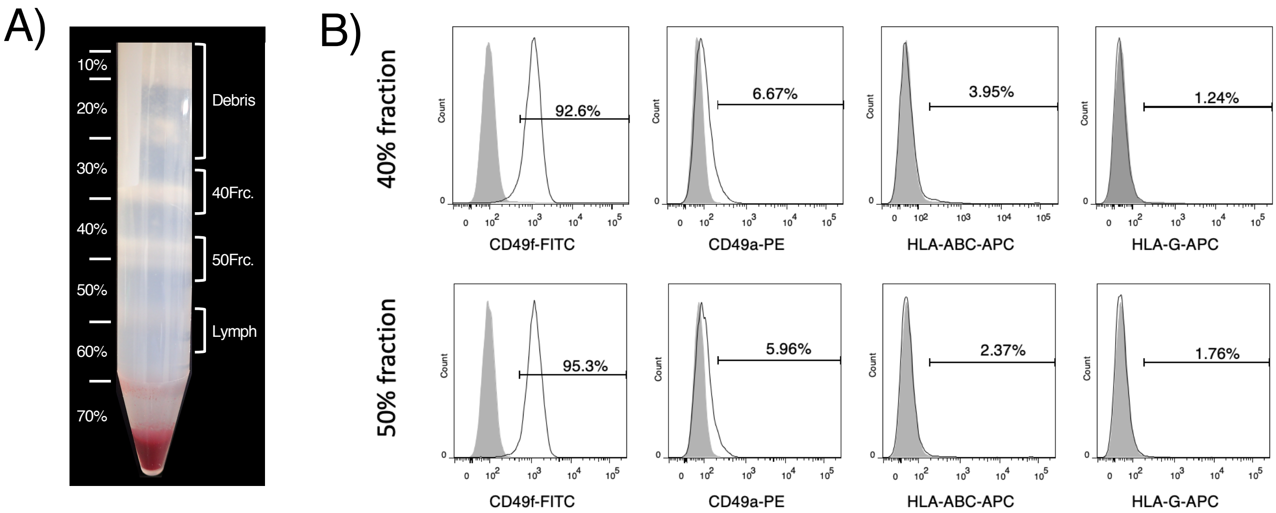


**Supplementary Figure S6. Isolation of primary villous trophoblast cells**

A) Placental lysates separated across a Percoll gradient contain trophoblast cells in the 40% and 50% fractions. B) Cells from these fractions were subjected to flow cytometry using trophoblast specific markers and antibodies against HLA-ABC and HLA-G to assess trophoblast cell purity.

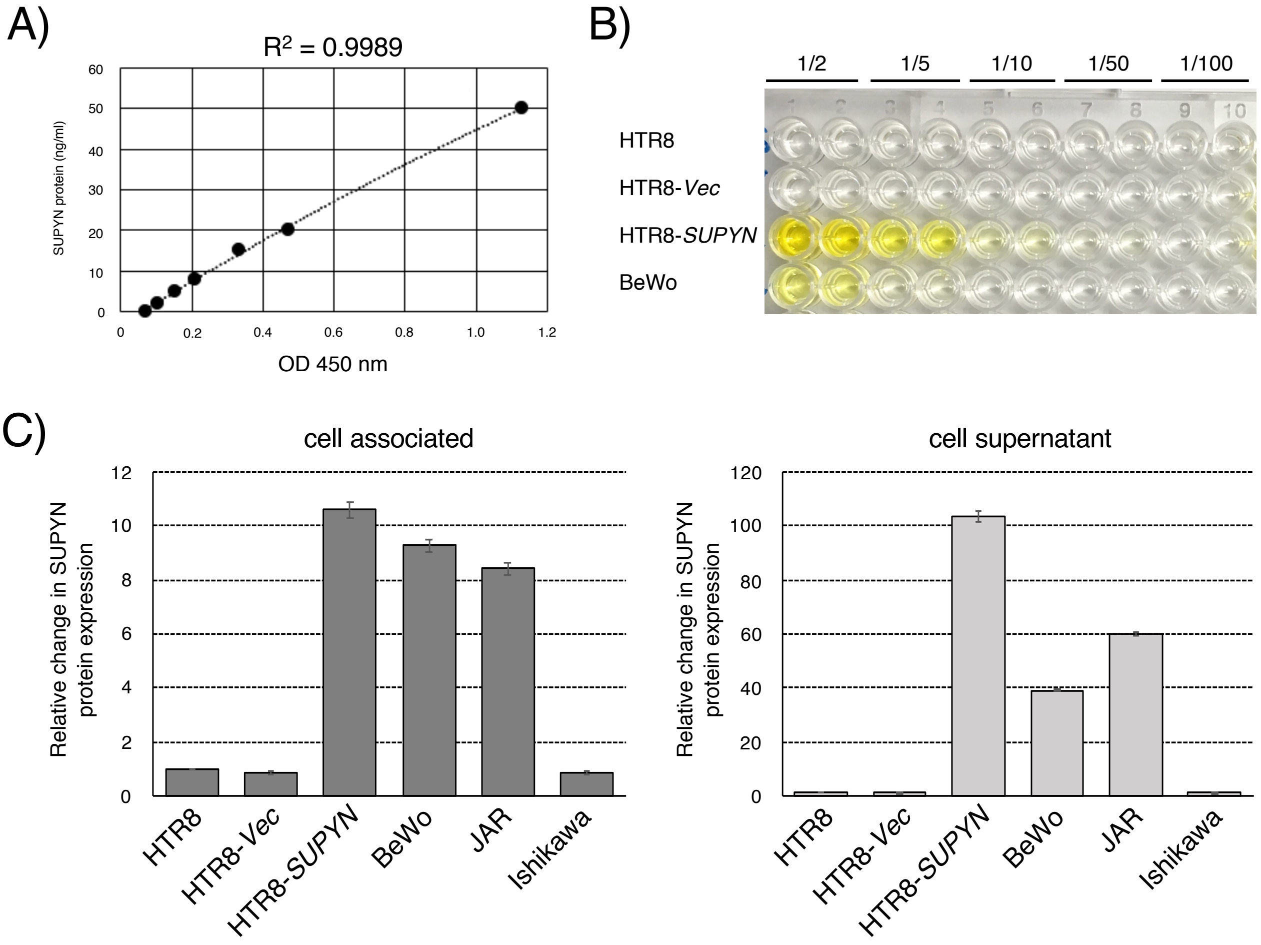


**Supplementary Figure S7. A SUPYN-specific ELISA was developed using two anti-SUPYN monoclonal antibodies.**

A) Dilution linearity validation testing was performed using a synthetic 50aa SUPYN peptide (111-160). B) ELISA specificity was confirmed using endogenous SUPYN-expressing BeWo trophoblast cells and stably transfected SUPYN-expressing HTR8 cells. Cell supernatant sample dilution series (1/2 – 1/100). C) Negative control (HTR8, Ishikawa endometrial cell) and positive control (BeWo, JAR, stably transfected SUPYN expressing HTR8) cells were lysed in RIPA buffer and diluted samples assayed. Undiluted cell supernatants were used. Data represent relative fold change compared with HTR8 cell associated or supernatant samples, respectively. Values represent means ± SDs (n = 4).

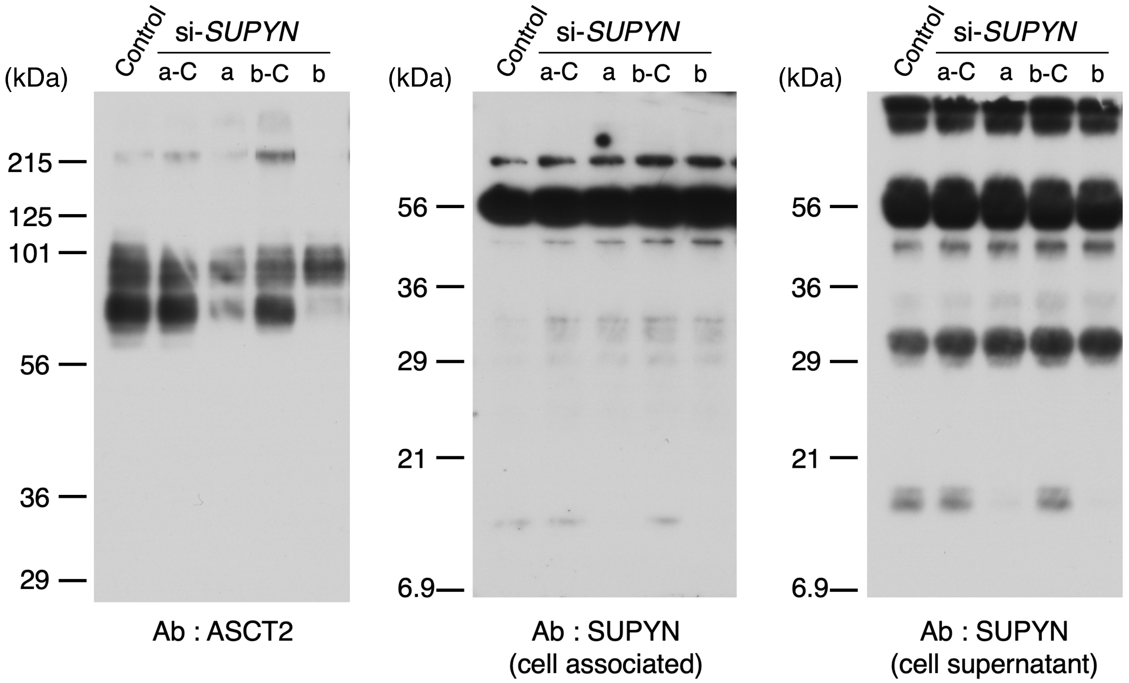


**Supplementary Figure S8. Association between SUPYN and ASCT2 induces glycosylation changes; full immunoblot for Figure 2B.**

Structural changes in ASCT2 protein upon knockdown of SUPYN in BeWo cells using suppressyn-specific siRNAs. Exposures to siRNAs a and b and their scrambled controls (a-C and b-C, respectively) and a vector only control (Control) are shown.

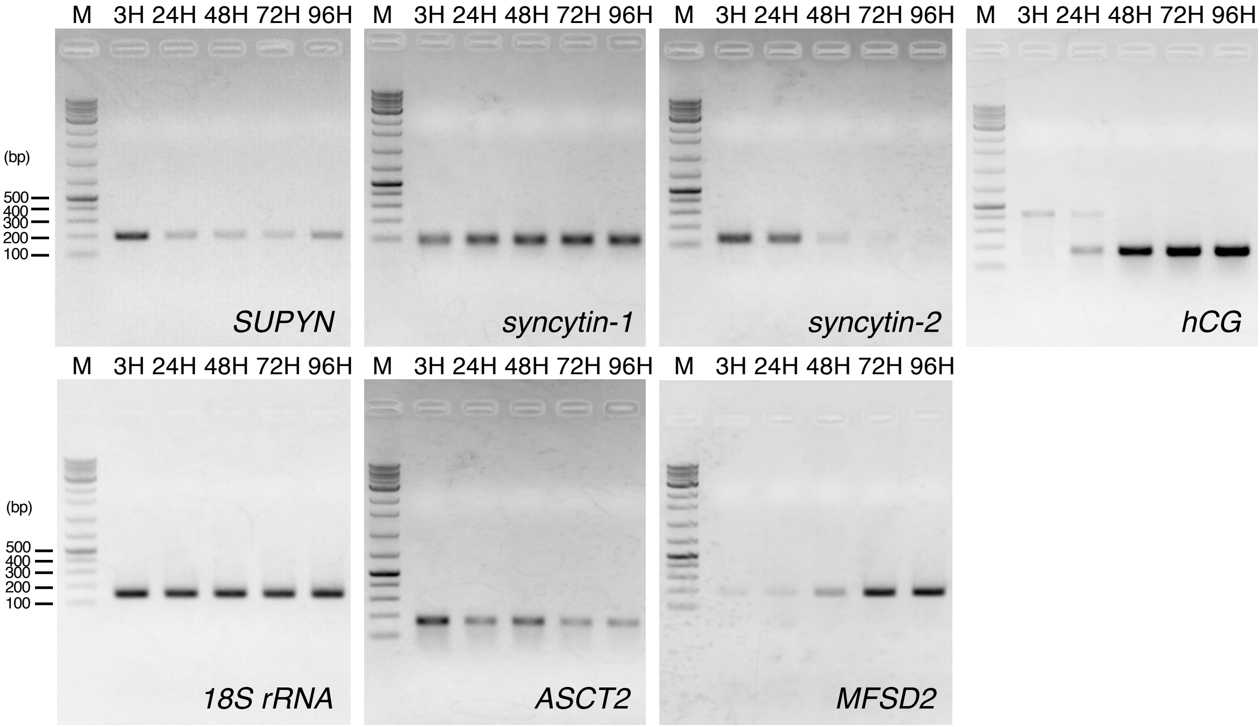


**Supplementary Figure S9. Cell fusion and expression of placental fusion-related proteins and hormones in primary spontaneously syncytializing peri-term human cytotrophoblast cells with and without forced SUPYN expression ; full gel image for Figure 3A.**

Expression of fusion-associated genes in primary trophoblast cells using semi-quantitative RT-PCR. M ; marker. *SUPYN*- suppressyn; *ASCT2*-- Alanine, Serine, Cysteine Transporter 2 , the syncytin-1 receptor; *MFSD2*-- major facilitator superfamily domain-containing protein 2, the syncytin-2 receptor; *hCG*-- human chorionic gonadotropin.

| | Species | Clone No. | Cat No. | Distributor | Conc. | Dilution | | | |
|---|---|---|---|---|---|---|---|---|---|
| | | | | | | IHC | ICC | WB | FACS |
| Suppressyn | Mouse | 3H6 | na | na | 75ng/μl | 1/250 | 1/500 | 1/3000 | |
| | | 2J16 | | | 45ng/μl | | | | |
| ASCT2 | Rabbit | D7C12 | 8057 | CST | 55ng/ml | 1/1000 | 1/1000 | 1/2000 | |
| Syncytin-1 | Rabbit | na | ab71115 | abcam | 0.25mg/ml | 1/2000 | | | |
| HLA-G(MEM-G/1) | Mouse | MEM-G/1 | ab7759 | abcam | 1mg/ml | 1/6000 | 1/500 | | |
| Cytokeratin7 | Rabbit | na | ab81074 | abcam | na | 1/8000 | | | |
| β-actin | Mouse | AC-15 | A5441 | Sigma | 26mg/ml | | | 1/10000 | |
| E-cadherin | Mouse | HECD-1 | ab1416 | abcam | na | | 1/500 | | |
| Anti-DDDDK-tag | Mouse | FLA-1 | M185 | MBL | 1mg/ml | | 1/1000 | 1/3000 | |
| ZO-1/TJP1 | Mouse | ZO1-1A12 | 33-9100 | Thermo Scientific | 0.5mg/ml | | 1/500 | | |
| Mouse IgG isotype | Mouse | na | 02-6502 | Thermo Scientific | 2.5mg/ml | depend on Ab conc. | | | |
| Rabbit mAb IgG XP isotype | Rabbit | DA1E | 3900 | CST | 2.5mg/ml | | | | |
| Alexa Fluor 488 goat anti-mouse IgG(H+L) | Mouse | na | A11029 | Thermo Scientific | 2mg/ml | | 1/1000 | | |
| Alexa Fluor 555 goat anti-rabbit IgG(H+L) | Rabbit | na | A21429 | Thermo Scientific | 2mg/ml | | 1/1000 | | |
| Anti-mouse IgG HRP-linked Antibody | Mouse | na | 7076 | CST | na | | | 1/3000 | |
| Anti-rabbit IgG HRP-linked Antibody | Rabbit | na | 7074 | CST | na | | | 1/3000 | |
| FITC anti-human CD49f | Rat | GoH3 | 313605 | BioLegend | na | | | | 3μl |
| PE anti-human CD49a | Mouse | TS2/7 | 328303 | BioLegend | na | | | | 3μl |
| APC anti-human-HLA-A,B,C | Mouse | W6/32 | 311409 | BioLegend | na | | | | 3μl |
| APC anti human-HLA-G | Mouse | 87G | 335909 | BioLegend | na | | | | 3μl |
| FITC Rat IgG2a | Rat | RTK2758 | 400505 | BioLegend | 0.5mg/ml | | | | 3μl |
| PE Mouse IgG1 | Mouse | MOPC-21 | 400111 | BioLegend | 0.2mg/ml | | | | 3μl |
| APC Mouse IgG2a | Mouse | MOPC-173 | 400219 | BioLegend | 0.2mg/ml | | | | 3μl |

**Supplementary Table S1. Antibodies used for immunohistochemistry, immunofluorescence imaging and flow cytometry.**

| *Gene* | Primer ID | Sequence |
|---|---|---|
| RT-PCR | | |
| *SUPYN* | Fb1-S | TCCGGGTTCCAACCAATGCAAGA |
| | Fb1-AS | TGTGCCAGTAGGCGAGATCAGT |
| *syncytin-1* | Syn1-S | CCACGAACGGACATCCAA |
| | Syn1-AS | TCCACTCCAGCCACTTTAAC |
| *ASCT2* | ASCT2-S | TCGATTCGTTCCTGGATCTTGCGA |
| | ASCT2-AS | ACACTACCAAGCCCAGGATGTTCA |
| *syncytin-2* | Syn2-S | TCTCAAATGGTGCAGTGACTCGGA |
| | Syn2-AS | TGCTGGTTCTGGCTCTGGAGTTTA |
| *MFSD2* | MFSD2-S | TCGCCTTATGCCCTGGATCATCTT |
| | MFSD2-AS | TCGGTGCTGATGAACATGGTGAGA |
| *hCG* | hCG－S | CATCACCGTCAACACCACCATCT |
| | hCG－AS | AGGAGACCACGGGGTTCACG |
| *18S rRNA* | 18S rRNA-S | GTAACCCGTTGAACCCCATT |
| | 18S rRNA-AS | CCATCCAATCGGTAGTAGCG |
| Cloning | | |
| *ZAG* | ZAG-S-EcoRV | CGGATATCAA GAATGGTGCC TGTCCTGCT |
| | ZAG-AS-BamHI | CCGGATCCTC GCTGGCCTCC CAGGGCA |
| Mutagenesis | | |
| *ASCT2-N163H* | 163-S | GCCATCCACGCCTCCGTGGGAGCCGCG |
| | 163-AS | CCTCCGCACCTACCGCCGCCTCCGCCG |
| *ASCT2 -N212H* | 212-S | GAGAGGCACATCACCGGAACCAGGGTG |
| | 213-AS | CCACTACACGGAGAGAAGTATCCACCA |

**Supplementary Table S2. Primers used for gene cloning, mutagenesis, and RT-PCR** *SUPYN*- suppressyn; *ASCT2*-- Alanine, Serine, Cysteine Transporter 2 , the syncytin-1 receptor; *MFSD2*-- major facilitator superfamily domain-containing protein 2, the syncytin-2 receptor; *hCG*-- human chorionic gonadotropin.
